# High Molecular Weight Barley β-Glucan Alters Gut Microbiota Toward Reduced Cardiovascular Disease Risk

**DOI:** 10.3389/fmicb.2016.00129

**Published:** 2016-02-10

**Authors:** Yanan Wang, Nancy P. Ames, Hein M. Tun, Susan M. Tosh, Peter J. Jones, Ehsan Khafipour

**Affiliations:** ^1^Department of Human Nutritional Sciences–Richardson Centre for Functional Foods and Nutraceuticals, University of Manitoba, WinnipegMB, Canada; ^2^Cereal Research Center, Agriculture and Agri-Food Canada, WinnipegMB, Canada; ^3^Gut Microbiome Laboratory, Department of Animal Science, University of Manitoba, WinnipegMB, Canada; ^4^Guelph Research and Development Center, Agriculture and Agri-Food Canada, GuelphON, Canada; ^5^Department of Medical Microbiology, University of Manitoba, WinnipegMB, Canada

**Keywords:** gut microbiota, β-glucan, molecular weight, cardiovascular disease, 16S rRNA gene sequencing

## Abstract

The physiological cholesterol-lowering benefits of β-glucan have been well documented, however, whether modulation of gut microbiota by β-glucan is associated with these physiological effects remains unknown. The objectives of this study were therefore to determine the impact of β-glucan on the composition of gut microbiota in mildly hypercholesterolemic individuals and to identify if the altered microbiota are associated with bioactivity of β-glucan in improving risk factors of cardiovascular disease (CVD). Using a randomized, controlled crossover study design, individuals received for 5-week either a treatment breakfast containing 3 g high molecular weight (HMW), 3 g low molecular weight (LMW), 5 g LMW barley β-glucan, or wheat and rice. The American Heart Association (AHA) diet served as the background diet for all treatment groups. Phases were separated by 4-week washout periods. Fecal samples were collected at the end of each intervention phase and subjected to Illumina sequencing of 16S rRNA genes. Results revealed that at the phylum level, supplementation of 3 g/d HMW β-glucan increased Bacteroidetes and decreased Firmicutes abundances compared to control (*P* < 0.001). At the genus level, consumption of 3 g/d HMW β-glucan increased *Bacteroides* (*P* < 0.003), tended to increase *Prevotella* (*P* < 0.1) but decreased *Dorea* (*P* < 0.1), whereas diets containing 5 g LMW β-glucan and 3 g LMW β-glucan failed to alter the gut microbiota composition. *Bacteroides, Prevotella*, and *Dorea* composition correlated (*P* < 0.05) with shifts of CVD risk factors, including body mass index, waist circumference, blood pressure, as well as triglyceride levels. Our data suggest that consumption of HMW β-glucan favorably alters the composition of gut microbiota and this altered microbiota profile associates with a reduction of CVD risk markers. Together, our study suggests that β-glucan induced shifts in gut microbiota in a MW-dependent manner and that might be one of the underlying mechanisms responsible for the physiological benefits of β-glucan.

## Introduction

Mixed-linkage (1→3)(1→4)-β-D-glucan (β-glucan) is a linear polysaccharide composed of D-glucose monomers joined by (1→4)-glycosidic bonds (∼70%) with an occasional (1→3)-glycosidic bonds (∼30%; Wood et al., 1991). β-glucan is the major soluble fiber in oat and barley (Johansson et al., 2008) and has shown to have physiological benefits including plasma cholesterol lowering (AbuMweis et al., 2010a; Whitehead et al., 2014), reduction of postprandial glycaemic responses (Braaten et al., 1991; Tappy et al., 1996; Jenkins et al., 2002), and weight management via increasing satiety (Maki et al., 2010; Cook et al., 2014). Previous investigations into the mechanisms underlying these beneficial effects are limited to the action of β-glucan in the upper gastrointestinal (GI) tract; however, since humans lack enzymes for the digestion of β-glucan and other dietary fibers, the majority of these compounds are fermented in the lower GI tract via carbohydrate active enzymes (CAZymes) produced by gut microbiota, potentially resulting in compositional and functional shifts in the microbiome (Cantarel et al., 2012; Chassard and Lacroix, 2013).

Gut microbiota have long been recognized to contribute to host nutrition and energy metabolism via production of short chain fatty acids, amino acids, and vitamins (Morowitz et al., 2011). The microbial-derived metabolites can be distributed well beyond the GI tract and influence the physiology of the host, enabling this complex network of microorganisms to act as a virtual endocrine organ, hence, playing an important role in host health and disease status (Fujimura et al., 2010; Sekirov et al., 2010). Altered gut microbiota has been associated with metabolic disorders, including obesity (Ley et al., 2006; Ley, 2010; Sanz et al., 2010; Shen et al., 2013), diabetes (Karlsson et al., 2013; Shen et al., 2013) and cardiovascular disease (CVD; Wang et al., 2011; Karlsson et al., 2012; Koeth et al., 2013). Current reports suggest that manipulation of gut microbiota toward enhancing the proportion of beneficial members of the community is a promising strategy for prevention or treatment of metabolic diseases (Fujimura et al., 2010; Karlsson et al., 2013; Shoaie et al., 2013). Mixed-linkage β-glucan as a fermentable dietary fiber (Rose and Hamaker, 2011) could actively impact the microbiota as exhibited from *in vitro* fermentation (Hughes et al., 2008) and *in vivo* intervention studies (Pieper et al., 2008; Mitsou et al., 2010). However, conclusions with regard to the impact of β-glucan on the microbiota composition and function are still obscure due to inconsistencies in study design, experimental models and most importantly methodologies used for bacterial community analyses. A well-designed clinical trial and an appropriate methodology for global bacterial community analysis, thus are required to provide comprehensive information concerning quantification of the shifts in the gut microbiota following β-glucan consumption in humans.

It is important to note that physiochemical properties of β-glucan, such as molecular weight (MW), are considered to be critical factors in its physiological properties (Wood, 2002; Wolever et al., 2010). The extent of β-glucan fermentation in the distal GI tract may also depend on its physiochemical structure (Hughes et al., 2008). Yet, whether β-glucan with low or high MW alters gut microbiota in a differential manner is unknown. In this study, we aimed to investigate whether consumption of barley β-glucan alters the composition of gut microbiota as represented by the feces (Krause and Khafipour, 2010; Santiago et al., 2014) and whether the alteration is in a MW dependent manner. Also, we aimed to predict shifts in the functional properties of the gut microbiome following barley β-glucan consumption and to examine whether shifts in gut microbiome composition associate with physiological effects resulting from β-glucan consumption.

## Materials and Methods

A randomized, single-blinded, controlled crossover trial was conducted at the Clinical Nutrition Research Unit at the Richardson Centre for Functional Foods and Nutraceuticals (RCFFN), University of Manitoba (Winnipeg, MB, Canada) in accordance with the principles expressed in the Declaration of Helsinki. All research procedures were approved by the University of Manitoba Biomedical Research Ethics Board (Ethic reference no. B2010:057). The clinical trial was registered with ClinicalTrials.gov (ID NCT01408719). The endpoint data for body weight, BMI, waist circumference, blood glucose and lipid profiles, and blood pressure for the 19 subjects included in this microbiome study extracted from the parallel clinical trial that focused on cholesterol lowering effect of β-glucan (Wang et al., 2016) and were reinterpreted in the context of their correlations with microbial changes evaluated in this research.

### Subjects

Male and female subjects aged 27–78 years (*n* = 30) with a body mass index (BMI; in kg/m^2^) between 20 and 40 were selected on the basis of fasting serum total cholesterol (TC) between 5.0 and 8.0 mmol/L and LDL cholesterol (LDL-C) between 2.7 and 5.0 mmol/L. Potential volunteers were excluded using criteria including: intake of blood lipid lowering medications, herbal or nutritional supplements which are known to affect blood lipids and any dietary restrictions which would affect trial completion. No antibiotics intake was reported at the time of screening. A study physician evaluated the health history of volunteers before they were accepted as subjects. The physician also provided medical supervision throughout the study. Written informed consent was obtained from all subjects. Of the 30 subjects in this study, a subset of 19 subjects who completed the entire protocol and provided fecal samples for all four study phases were selected for the microbiota profile analysis.

### Study Design and Experimental Diets

As described in the parallel study (Wang et al., 2016), the human clinical trial consisted of four 5-week study phases separated by 4-week washout periods. Subjects were randomized to one of four experimental diets in each phase: (a) a wheat and rice (WR) based control; (b) 3 g low molecular weight (LMW) barley β-glucan (288 kDa); (c) 5 g LMW barley β-glucan (292 kDa); and (d) 3 g high molecular weight (HMW) barley β-glucan (1,349 kDa). Each subject received all four treatments during the study in randomized orders. The barley treatments were incorporated into breakfast in the format of crepes, tortillas, porridge, and chips. WR were used to replace barley in breakfast as a control. Lunch and dinner were designed using a 7-day rotating menu to provide daily energy needs. Energy requirements of each subject were estimated based on age, gender, body weight, and physical activity levels and adjusted proportionately in 300 kcal increments. Macronutrient profiles of treatment diets provided approximately 30% of energy as fat, 55% as carbohydrate, and 15% as protein. Subjects consumed their intervention breakfast meals under supervision at the clinical unit at the RCFFN on weekdays to ensure compliance. Remaining weekday and weekend meals including intervention breakfast were packed for home consumption and empty containers were returned to ensure compliance.

Body weight was monitored everyday on weekdays at the clinical unit prior to breakfast. Waist circumference was measured at the beginning and at the end of each intervention phase. Fasting serum and plasma samples were collected on days 1, 2, 34 and 35. Serum TC, HDL cholesterol (HDL-C), LDL-C, and triglyceride (TG) levels were measured. Approximately 15 g of stool samples were collected at the end of each phase between days 30 and 35. Subjects were advised to collect stool samples from three locations of one bowel movement and immediately store the collected samples in their household –20°C freezer. Upon transport, samples were transferred to chilled coolers and then stored for future analysis at –80°C.

### DNA Extraction and Quality Check

Stool samples from different phases of the projects were DNA extracted and sequenced at once. For DNA extraction, approximately 200 mg of each fecal sample was used for DNA extraction using ZR Fecal DNA MiniPrep^TM^ (Zymo Reseach, Irvine, CA, USA), which included a bead-beating step for the mechanical lysis of the microbial cells. DNA was quantified using a NanoDrop 2000 spectrophotometer (Thermo Scientific, Waltham, MA, USA). DNA samples were normalized to 20 ng/μl, and quality checked by PCR amplification of the 16S rRNA gene using universal primers 27F (5′-GAAGAGTTTGATCATGGCTCAG-3′) and 342R (5′-CTGCTGCCTCCCGTAG-3′) as described by Khafipour et al. (2009). Amplicons were verified by agarose gel electrophoresis.

### Library Construction and Illumina Sequencing

Library construction and Illumina sequencing were performed as described by Derakhshani et al. (2015). In brief, the V4 region of 16S rRNA gene was targeted for PCR amplification using modified F515/R806 primers (Caporaso et al., 2012). The reverse PCR primer was indexed with 12-base Golay barcodes allowing for multiplexing of samples. PCR reactions were performed in duplicate for each sample and contained 1.0 μl of pre-normalized DNA, 1.0 μl of each forward and reverse primers (10 μM), 12 μl HPLC grade water (Fisher Scientific, Ottawa, ON, Canada) and 10 μl 5 Prime Hot MasterMix^®^ (5 Prime, Inc., Gaithersburg, MD, USA). Reactions consisted of an initial denaturing step at 94°C for 3 min followed by 35 amplification cycles at 94°C for 45 s, 50°C for 60 s, and 72°C for 90 s; finalized by an extension step at 72°C for 10 min in an Eppendorf Mastercycler^®^ pro (Eppendorf, Hamburg, Germany). PCR products were then purified using a ZR-96 DNA Clean-up Kit^TM^ (ZYMO Research, Irvine, CA, USA) to remove primers, dNTPs and reaction components. The V4 library was then generated by pooling 200 ng of each sample, quantified by Picogreen dsDNA (Invitrogen, Burlington, ON, Canada). This was followed by multiple dilution steps using pre-chilled hybridization buffer (HT1; Illumina, San Diego, CA, USA) to bring the pooled amplicons to a final concentration of 5 pM, measured by an Qubit^®^ 2.0 Fluorometer (Life technologies, Burlington, ON, Canada). Finally, 15% of PhiX control library was spiked into the amplicon pool to improve the unbalanced and biased base composition, a known characteristic of low diversity 16S rRNA libraries. Customized sequencing primers for read1 (5′-TATGGTAATTGTGTGCCAGCMGCCGCGGTAA-3′), read2 (5′-AGTCAGTCAGCCGGACTACHVGGGTWTCTAAT-3′) and index read (5′-ATTAGAWACCCBDGTAGTCCGGCTGACTGACT-3′) were synthesized and purified by polyacrylamide gel electrophoresis (Integrated DNA Technologies, Coralville, IA, USA) and added to the MiSeq Reagent Kit v2 (300-cycle; Illumina, San Diego, CA, USA). The 150 paired-end sequencing reaction was performed on a MiSeq platform (Illumina, San Diego, CA, USA) at the Gut Microbiome and Large Animal Biosecurity Laboratories, Department of Animal Science, University of Manitoba, Canada. All sequencing data are uploaded into the Sequence Read Archive (SRA) of NCBI and are available through accession number SRR2558312.

### Bioinformatic Analyses

Bioinformatic analyses were performed as described by Derakhshani et al. (2015). In brief, the PANDAseq assembler (Masella et al., 2012) was used to merge overlapping paired-end Illumina fastq files. Sequences with mismatches or ambiguous calls in the overlapping region were discarded. The output fastq file was then analyzed by downstream computational pipelines of the open source software package QIIME (Caporaso et al., 2010b). Assembled reads were de-multiplexed according to the barcode sequences and exposed to additional quality-filters so that reads with more than three consecutive bases with quality scores below 1*e* – 5 were truncated, and those with a read length shorter than 75 bases were removed from the downstream analysis. Chimeric reads were filtered using UCHIME (Edgar et al., 2011) and sequences were assigned to operational taxonomic units (OTU) using the QIIME implementation of UCLUST (Edgar, 2010) at 97% pairwise identity threshold. Taxonomies were assigned to the representative sequence of each OTU using RDP classifier (Wang et al., 2007) and aligned with the Greengenes Core reference database (DeSantis et al., 2006) using PyNAST algorithms (Caporaso et al., 2010a). The phylogenetic tree was built with FastTree 2.1.3. (Price et al., 2010) for further comparisons between microbial communities. Finally, the open source software PICRUSt (Phylogenetic Investigation of Communities by Reconstruction of Unobserved States, version 1.0.0) was used to predict metagenome function based on 16S rRNA gene sequences (Langille et al., 2013). For the PICRUSt analysis, closed-reference OTUs were picked at 97% similarity against the Greengenes database. The OTUs were normalized for predicted 16S rRNA copy number before predicting gene family abundance for each metagenome based on KEGG orthology groups (KOs) using the Kyoto Encyclopedia of Genes and Genomes (Langille et al., 2013).

### Alpha- and β-Diversity Measurements

Within community diversity (α-diversity) was calculated using QIIME. The α rarefaction curve was generated using Chao1 estimator of species richness (Chao, 1984) with 10 sampling repetitions at each sampling depth. An even depth of approximately 3,984 sequences per sample was used for calculation of richness and diversity indices. To compare microbial composition between samples, β-diversity was measured by calculating the weighted and unweighted UniFrac distances (Lozupone and Knight, 2005) using QIIME default scripts. Principal coordinate analysis (PCoA) generated two-dimensional plots using PRIMER v6 software using resulting distance matrices (Clarke and Gorley, 2006).

### Statistical Analysis

Statistical analyses were performed as described by Li et al. (2012) and Derakhshani et al. (2015). Non-normally distributed data were Box–Cox transformed and then used to assess the effect of experimental diet using MIXED procedure of SAS version 9.2 (SAS Institute Inc., Cary, NC, USA). Changes of α-diversity indices (Chao1, Good’s coverage, Shannon and Simpson, Observed species) were tested using a MIXED procedure of SAS. Differential abundances in bacterial phyla and genera were tested using MIXED and GLIMMIX procedures of SAS, with treatment, phase and phase × treatment as fixed factors. For non-normally distributed data, Poisson and negative binomial distributions were fitted in the GLIMMIX procedure of SAS. A log link function was specified for Poisson and negative binomial distributions. The goodness of fit for each distribution was compared using Pearson chi-square/DF value (closer to 1 is better) and used for selection of best distribution. Subjects were treated as a random factor and the correlation between repeated measures of these individuals was modeled using the first-order autoregressive (AR-1) option. Pairwise comparisons among the groups were tested using Tukey studentized range adjustment. The differences between groups were considered significant at *P* < 0.05 while trends were observed at *P* < 0.1.

Permutational multivariate analysis of variance (PERMANOVA; Anderson, 2005) was used to calculate *P*-values and test for significant differences of β-diversity among treatment groups. The effects of treatment, period, and treatment by period interaction were considered fixed factors and subject as the random factor.

Partial least square discriminant analysis (PLS-DA; SIMCA P+ 13.0, Umetrics, Umea, Sweden) was performed on genus data to identify the effects of treatments (**Figure 2**). The PLS-DA is a particular case of partial least square regression analysis in which Y is a set of variables describing the categories of a categorical variable on X. In this case, X variables were bacterial genera and Y variables were experimental diets. For this analysis, data were scaled using Unit Variance in SIMCA. Cross-validation was then performed to determine the number of significant PLS components and a permutation testing was conducted to validate the model. To avoid over-parameterization of the model, variable influence on projection value (VIP) was estimated for each genus and genera with VIP < 0.50 were removed from the final model (Pérez-Enciso and Tenenhaus, 2003; Verhulst et al., 2011). An *R*^2^ estimate then was used to evaluate the goodness of fit and a *Q*^2^ estimate was used to evaluate the predictive value of the model. The PLS-regression coefficients were used to identify genera that were most characteristics of each treatment group. The significant shifts of taxa were determined when the error bars of each component was above or below x axis of coefficient plot. The results of PLS-DA were visualized by PLS-DA loading scatter plots. **Figure 2** presents the PLS-DA comparisons only between those treatments that were significantly different in β-diversity.

Welch’s *t*-test was applied on gene function from PICRUSt outputs, percentage of the predicted metagenome made by a given KEGG functional module, to compare differences in predicted metagenomic functions between treatment groups.

Correlations between bacterial taxa and clinical parameters were performed using non-parametric Spearman’s rank correlation (JMP, Version 10; SAS Institute Inc., Cary, NC, USA). The clinical parameters and bacterial taxa defined as response (Y) variables and a multivariate platform was used to examine how many variables relate to each other. For each correlation, the correlation coefficient (Spearman’s ρ) and *P*-value were obtained. The correlation coefficient values ranged from –1 to +1 with larger absolute values indicating stronger relationship while positive and negative values indicating the direction of association. Alpha value for the correlation confidence intervals was set up as 0.05.

## Results

### Changes in Metabolic Biomarkers

The results of blood glucose, blood pressure, body weight, waist circumference, and BMI were consistent with the results of parallel study that included 30 subjects and focused on examining the blood cholesterol lowering effects of β-glucan (Supplementary Table 1 and Wang et al., 2016). The only exception was the TC levels, which was reduced by 3 g HMW β-glucan in the parallel study but not in the sub-set of 19 individuals subjected to microbiome analysis.

### Changes in α- and β-Diversity

**FIGURE 1:**
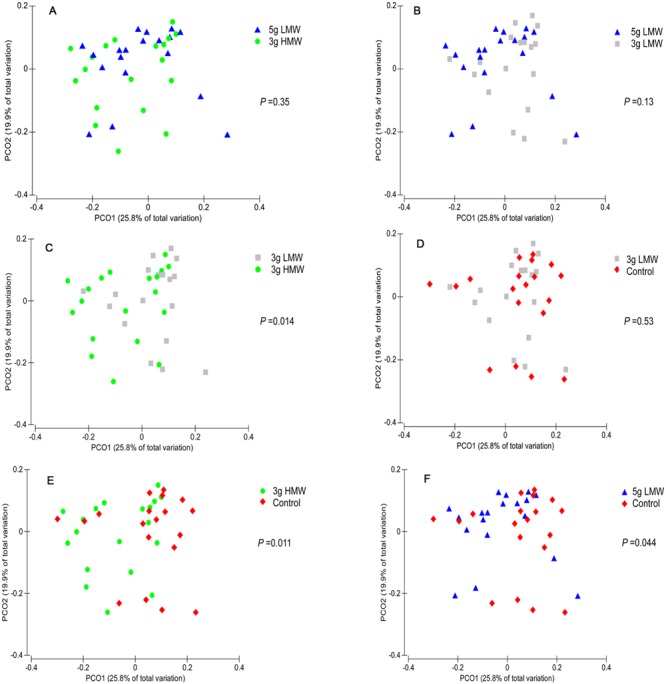
**Principal coordinates analysis (PCoA) of weighted UniFrac distances.** Between community diversity among stool samples was tested for the impact of β-glucan amount and MW on the clustering patterns of microbiota in **(A)** 5 g LMW vs. 3 g HMW (*P* = 0.35), **(B)** 3 g LMW vs. 3 g HMW (*P* = 0.13), **(C)** 3 g LMW vs. 3 g HMW (*P* = 0.014), **(D)** 3 g LMW vs. Control (*P* = 0.53), **(E)** 3 g HMW vs. Control (*P* = 0.011), **(F)** 5 g LMW vs. Control (*P* = 0.044). Symbols with different colors represent samples belonging to individuals under specific treatment. For each comparison, the *P*-value was obtained from PERMANOVA and considered significant for *P* < 0.05.

**FIGURE 2:**
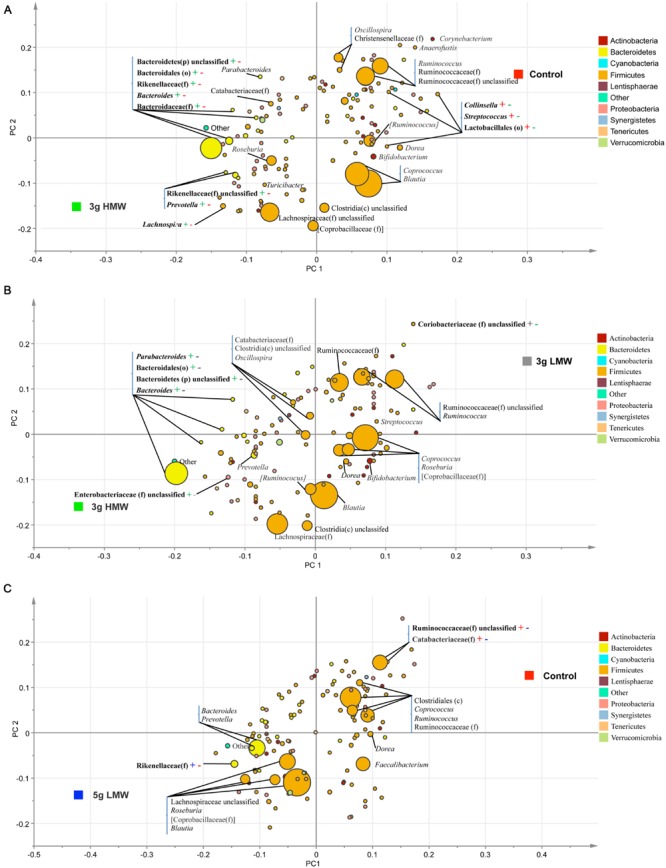
**Partial least square discriminant analysis (PLS-DA) loading plot.** The PLS-DA graphs were generated based on the relative abundance of the putative bacterial genera in stool samples and their association with **(A)** 3 g HMW or control diet, **(B)** 3 g HMW or 3 g LMW diet, and **(C)** 5 g LMW or control diet. – or + signs and their color are indicative of significant negative or positive associations between each taxa and the associated treatment.

Alpha-diversity indices of fecal microbiota, or the diversity within a community, did not change in response to consumption of β-glucan (**Table 1**). In contrast, β-diversity of the fecal microbial community, or the dissimilarity between communities, measured by weighted UniFrac was affected by β-glucan supplementation (*P* = 0.002, PERMANOVA, **Supplementary Figure 1**). In the pairwise comparison, significant differences were observed among microbial communities between 3 g HMW and 3 g LMW β-glucan (*P* = 0. 014, **Figure 1C**), 3 g HMW β-glucan and the control (*P* = 0. 011, **Figure 1E**), and 5 g LMW β-glucan and the control (*P* = 0. 044, **Figure 1F**). The differences between 5 g LMW and 3 g HMW β-glucan (**Figure 1A**), 5 g LMW and 3 g LMW β-glucan (**Figure 1B**), 3 g LMW β-glucan and control (**Figure 1D**) were not significant. The community composition profile of the above-mentioned three paired comparisons, which showed significant differences in their β-diversity, were further compared using PLS-DA as discussed below (**Figure 2**).

**Table 1:** Biodiversity of microbial communities in feces affected by β-glucan diets.

Alpha-diversity^1^	Diet^2^				SEM^3^	*P-*value^4^
	WR Control	3 g LMW	5 g LMW	3 g HMW		
Chao1	2502	2594	2548	2730	122	0.22
Good’s coverage	0.85	0.84	0.84	0.83	0.01	0.14
Observed species	865	893	881	931	33	0.16
Shannon	7.45	7.54	7.51	7.68	0.19	0.36
Simpson	0.98	0.98	0.98	0.98	0.003	0.58

*^1^Chao1 and Good’s coverage are the richness indices; Shannon and Simpson are diversity estimators.*

*^2^Diets include: Wheat and rice control (WR Control), 3 g/d low molecular weight barley β-glucan (3 g LMW), 5 g/d low molecular weight barley β-glucan (5 g LMW), 3 g/d high molecular weight barley β-glucan (3 g HMW). Data are presented as mean values.*

*^3^SEM, standard error of means.*

*^4^*P-*values are shown for the diet effect between groups analyzed by mixed model ANCOVA with period and the interaction of period × diet as fixed factors.*

### Compositional Shifts in Bacterial Communities

While majority of OTUs were identified at the genus level, some were only classified at the phylum (p.), class (c.), order (o.), or family (f.) levels. Across all samples, 11 bacterial phyla were identified of which two phyla Firmicutes and Bacteroidetes were abundant (above 1% of community), accounting for approximately 84 and 9% of the entire bacterial community, respectively. Another four bacterial phyla, including Actinobacteria, Verrucomicrobia, Proteobacteria, and Tenericute were present at abundances between 0.1 and 1% of the community (**Table 2**). The remaining five phyla including Cyanobacteria, Synergistetes, Lentisphaerae, Deferribacteres, and Elusimicrobia were in low abundance (<0.1% of community). Abundances of p. Firmicutes and Bacteroidetes were significantly impacted in response to 3 g/d HMW β-glucan consumption, where Firmicutes decreased (77.91% for 3 g HMW β-glucan vs. 89.69% for control, *P* < 0.001) and Bacteroidetes increased (14.22% for 3 g HMWβ-glucan vs. 4.99% for control, *P* < 0.001) compared to the control (**Table 2**). However, Firmicutes and Bacteroidetes abundances did not change in response to 5 g/d LMW and 3 g/d LMW β-glucan consumption. The abundances of p. Actinobacteria, Verrucomicrobia, Proteobacteria, and Tenericute did not change across all treatments.

**Table 2:** Relative abundances of bacterial phyla in feces after β-glucan intervention.

Phylum^1^	Diet^2^				SEM^3^	*P-*value
	Control	3 g LMW	5 g LMW	3 g HMW		
**Above 1 % of community**					
Bacteroidetes	4.99^a^	7.59^a^	9.95^ab^	14.22^b^	1.67	< 0.001^4^
Firmicutes	89.69^a^	87.50^a^	82.36^ab^	77.91^b^	2.16	< 0.001^4^
**Between 0.1 and 1% of community**					
Actinobacteria	0.64	0.71	1.07	0.55	0.25	0.33^5^
Proteobacteria	0.18	0.10	0.14	0.32	0.13	0.66^5^
Tenericutes	0.086	0.066	0.093	0.19	0.06	0.47^5^
Verrucomicrobia	0.14	0.39	0.19	0.39	0.17	0.28^5^

*^a,b^Means within the same row with different superscripts are different (*P* < 0.05).*

*^1^Only phyla with abundances greater than 0.1% of the community (at least in one of the treatment group) are presented. Additional phyla including Cyanobacteria, Synergistetes, Lentisphaerae, Deferribacteres, Elusimicrobia were present in abundances below 0.1% of the community.*

*^2^Diets include: Wheat and rice control (Control), 3 g/d low molecular weight barley β-glucan (3 g LMW), 5 g/d low molecular weight barley β-glucan (5 g LMW), 3 g/d high molecular weight barley β-glucan (3 g HMW). Data are presented as mean values.*

*^3^SEM, Standard Error of Mean.*

*^4^*P*-values are shown for the diet effect between groups analyzed by mixed model ANCOVA with diet, period, period × diet as fixed factors.*

*^5^*P*-values are shown for the diet effect between groups analyzed by GLIMMIX procedure of SAS with diet, period, period × diet as fixed factors.*

At the lower taxonomical level and across all samples, 169 distinct bacterial taxa were detected of which 44 had a relative abundance above 0.1% of community (**Table 3**). Of these 44 taxa, 24 were identified at the genus level, 14 at the family level, four at the order level, and two at the class level. Irrespective of treatment group, g. *Bacteroides* was the most abundant genera in p. Bacteroidetes, whereas p. Firmicutes were dominated by g. *Blautia*. Consumption of 3 g/d HMW β-glucan increased the abundances of *Bacteroides* (12.26% vs. 5.96%, *P* = 0.002) compared to control. Consumption of 3 g/d HMW β-glucan also increased the relative abundance of o. Bacteroidales compared to the 3 g LMW β-glucan treatment (0.24 vs. 0.12%, *P* = 0.04). Moreover, f. Rikenellaceae and *g. Prevotella* showed increasing trends (*P* < 0.1) following 3 g/d HMW β-glucan consumption compared to control (**Table 3**). In contrast, *g. Streptococcus* (p. Firmicutes) was significantly decreased by 3 g/d HMW β-glucan compared to control (0.035% vs. 0.27%, *P* = 0.02). Additionally, g. *Dorea* showed a decreasing trend (*P* < 0.1) in response to 3 g/d HMW β-glucan consumption (**Table 3**).

**Table 3:** Relative abundances of bacterial genera in feces after β-glucan intervention.

Phyla	Taxa^1^	Diet^2^				SEM^3^	*P-*value
		Control	3 g LMW	5 g LMW	3 g HMW		
**Actinobacteria**	g. *Bifidobacterium*	1.03^3^	1.00	1.24	0.67	0.30	0.57^4^
**Bacteroidetes**							
	g. *Bacteroides*	5.96^a^	6.31^ab^	8.30^ab^	12.26^b^	1.60	0.002^4^
	f. Rikenellaceae	0.75	1.15	1.24	1.76	0.41	0.05^5^
	g. *Prevotella*	0.48	0.54	1.46	1.84	0.71	0.09^5^
	f. [Barnesiellaceae]	0.11	0.2	0.31	0.57	0.19	0.37^5^
	g. *Parabacteroides*	0.17	0.14	0.18	0.23	0.054	0.62^5^
	o. Bacteroidales	0.17^ab^	0.12^a^	0.21^ab^	0.24^b^	0.065	0.04^5^
	f. Bacteroidaceae	0.079	0.13	0.12	0.15	0.14	0.32^5^
**Firmicutes**							
	g. *Blautia*	11.43	9.31	11.55	8.5	1.78	0.35^4^
	g. *Coprococcus*	9.11	9.17	8.21	7.95	1.51	0.83^4^
	g. *Ruminococcus*	6.18	6.83	5.82	4.8	0.92	0.21^4^
	f. Lachnospiraceae unclassified	6.77	6.48	7.22	7.29	0.84	0.72^4^
	f. Ruminococcaceae	4.63	4.32	3.34	4.04	0.78	0.41^4^
	g. *Faecalibacterium*	5.34	4.71	3.92	4.32	1.57	0.17^5^
	f. Ruminococcaceae	3.12	2.85	1.97	2.98	0.82	0.50^4^
	f. Coprobacillaceae	2.45	2.91	3.31	2.91	0.66	0.55^4^
	g. *Roseburia*	1.75	2.73	2.74	2.26	0.55	0.28^4^
	g. [*Ruminococcus*]	3.43	2.86	2.99	2.76	0.58	0.49^4^
	g. *Oscillospira*	1.56	1.62	1.29	1.48	0.48	0.82^4^
	c. Clostridia unclassified	2.91	2.64	2.87	2.58	0.30	0.63^4^
	o. Clostridiales	1.28	1.24	1.06	1.3	0.20	0.70^4^
	g. *Dialister*	0.46	0.3	0.23	0.24	0.21	0.53^5^
	f. Christensenellaceae	0.05	0.33	0.14	0.17	0.17	0.77^5^
	g. *Dorea*	1.01	0.81	0.77	0.65	0.14	0.07^4^
	g. *Lachnospira*	0.4	0.46	0.36	1.06	0.22	0.18^5^
	f. Peptostreptococcaceae	0.34	0.67	0.47	0.26	0.19	0.47^4^
	f. Lachnospiraceae	0.43	0.43	0.31	0.69	0.19	0.54^4^
	f. Catabacteriaceae	0.42	0.61	0.35	0.81	0.50	0.27^4^
	g. *Catenibacterium*	0.3	0.56	0.73	0.35	0.37	0.23^4^
	c. Clostridia	0.14	0.17	0.13	0.17	0.04	0.63^4^
	o. Clostridiales unclassified	0.098	0.27	0.28	0.11	0.14	0.86^4^
	g. [*Eubacterium*]	0.15	0.12	0.11	0.11	0.12	0.98^4^
	p. Firmicutes unclassified	0.25	0.24	0.26	0.21	0.032	0.39^4^
	f. [Coprobacillaceae] unclassified	0.2	0.29	0.32	0.23	0.12	0.90^5^
	f. Clostridiaceae unclassified	0.11	0.12	0.091	0.15	0.09	0.97^5^
	g. *Clostridium*	0.097	0.14	0.084	0.12	0.08	0.96^5^
	g. *Turicibacter*	0.056	0.079	0.043	0.066	0.07	0.98^5^
	g. *Streptococcus*	0.27^a^	0.14^ab^	0.043^ab^	0.035^b^	0.07	0.02^5^
**Proteobacteria**					
	f. Enterobacteriaceae unclassified	0.12	0.011	0.18	0.54	0.19	0.09^5^
**Tenericutes**							
	o. RF39	0.082	0.062	0.085	0.17	0.058	0.52^5^
**Verrucomicrobia**							
	g. *Akkermansia*	0.14	0.38	0.19	0.39	0.17	0.28^5^

*^a,b^Means within the same row with different superscripts are different (*P* < 0.05).*

*^1^Some sequences were only affiliated to phylum (p.), class (c.), order (o.), family (f.), or genus (g.) levels.*

*^2^Diets include: Wheat and rice control (Control), 3 g/d low molecular weight barley β-glucan (3 g LMW), 5 g/d low molecular weight barley β-glucan (5 g LMW), 3 g/d high molecular weight barley β-glucan (3 g HMW). Data are presented as mean values.*

*^3^SEM, Standard Error of Mean.*

*^4^*P*-values are shown for the diet effect between groups analyzed by mixed model ANCOVA with diet, period, period × diet as fixed factors.*

*^5^*P*-values are shown for the diet effect between groups analyzed by GLIMMIX procedure of SAS with diet, period, period × diet as fixed factors.*

The PLS-DA results (given for the cut-off VIP value of 0.5) supported the SAS output, however, due to the higher sensitivity of PLS-DA methodology, additional significant differences among treatments, especially in low abundance taxa, were identified. For instance, when comparing 3 g HMW β-glucan vs. control, two additional low abundance taxa, g. *Collinsella* and o. Lactobacillales, were identified to be positively associated with control (**Figure 2A**).

### Correlations Between the Altered Bacterial Taxa and CVD Risk Factors

Spearman non-parametric correlation was conducted to test the association between bacterial taxa and risk factors of CVD (**Table 4**). Weak (Spearman’s ρ between 0.20 and 0.39) and moderate correlations (Spearman’s ρ between 0.40 and 0.59) were observed between parameters associated with CVD including BMI, waist circumference, TC levels, LDL-C, HDL-C, TG, glucose, systolic and diastolic blood pressure, and specific members of bacterial community (**Table 4**). Bacteria, which were affected by 3 g HMW β-glucan and also showed correlation with parameters associated with CVD, were g. *Bacteroides* which was negatively correlated with waist circumference (Spearman’s ρ = –0.25; *P* = 0.03) and diastolic BP (Spearman’s ρ = –0.28; *P* = 0.01); g. *Prevotella* which showed negative correlation with TG (Spearman’s ρ = -0.25; *P* = 0.02); and g. *Dorea* which was positively associated with BMI (Spearman’s ρ = 0.27; *P* = 0.01), waist circumference (Spearman’s ρ = 0.31; *P* = 0.008) and diastolic BP (Spearman’s ρ = 0.28; *P* = 0.01).

**Table 4:** Non-parametric correlations between metabolic parameters and bacterial taxa.

Metabolic parameters^1^	Taxa^2^	Phyla	Abundance^3^	Spearman’s ρ^4^	*P*-value^5^
BMI	g. *Turicibacter*	Firmicutes	0.16	-0.46	<0.0001
	g. *Catenibacterium*	Firmicutes	0.39	0.31	0.007
	f.[Barnesiellaceae]	Bacteroidetes	0.46	0.26	0.02
	f. Lachnospiraceae	Firmicutes	0.59	-0.21	0.07
	g. *Dorea*	Firmicutes	0.90	0.27	0.01
	f. Ruminococcaceae	Firmicutes	6.56	-0.28	0.01
	g. *Faecalibacterium*	Firmicutes	6.84	0.23	0.04
	g. *Ruminococcus*	Firmicutes	7.48	-0.27	0.01
	g. *Bacteroides*	Bacteroidetes	8.59	-0.20	0.08
Waist circumference	g. *Turicibacter*	Firmicutes	0.16	-0.47	<0.0001
	g. *Parabacteroides*	Bacteroidetes	0.19	-0.21	0.07
	g. [*Eubacterium*]	Firmicutes	0.27	0.27	0.02
	g. *Catenibacterium*	Firmicutes	0.39	0.25	0.03
	f. Lachnospiraceae	Firmicutes	0.59	-0.24	0.04
	g. *Dorea*	Firmicutes	0.90	0.31	0.008
	g. *Faecalibacterium*	Firmicutes	6.84	0.21	0.07
	g. *Bacteroides*	Bacteroidetes	8.59	-0.25	0.03
Total cholesterol	g. [*Ruminococcus*]	Firmicutes	3.38	-0.29	0.01
	g. *Roseburia*	Firmicutes	3.43	-0.24	0.04
	f. Ruminococcaceae	Firmicutes	6.56	0.24	0.03
	g. *Ruminococcus*	Firmicutes	7.48	0.32	0.005
LDL cholesterol	g. [*Eubacterium*]	Firmicutes	0.27	0.20	0.08
	g. [*Ruminococcus*]	Firmicutes	3.38	-0.33	0.004
	f. Ruminococcaceae	Firmicutes	6.56	0.29	0.01
	f. Lachnospiraceae unclassified	Firmicutes	7.37	-0.30	0.009
	g. *Ruminococcus*	Firmicutes	7.48	0.30	0.009
	g. *Blautia*	Firmicutes	11.27	-0.24	0.03
Triglyceride	f. Enterobacteriaceae Unclassified	Proteobacteria	0.18	0.25	0.03
	g. *Parabacteroides*	Bacteroidetes	0.19	-0.25	0.02
	f. Clostridiaceae unclassified	Firmicutes	0.24	0.34	0.002
	f. Catabacteriaceae	Firmicutes	0.43	-0.24	0.04
	f. Peptostreptococcaceae	Firmicutes	0.66	-0.28	0.01
	f. Christensenellaceae	Firmicutes	0.96	-0.38	0.0007
	g. *Dialister*	Firmicutes	1.06	0.25	0.03
	g. *Prevotella*	Bacteroidetes	1.09	-0.25	0.02
	g. *Oscillospira*	Firmicutes	3.02	-0.33	0.004
	f. Ruminococcaceae	Firmicutes	6.56	-0.26	0.02
	f. Lachnospiraceae unclassified	Firmicutes	7.37	0.24	0.03
HDL cholesterol	g. *Turicibacter*	Firmicutes	0.16	0.37	0.001
	g. [*Eubacterium*]	Firmicutes	0.27	-0.26	0.02
	g. *Dorea*	Firmicutes	0.90	-0.22	0.05
	g. *Dialister*	Firmicutes	1.06	-0.26	0.02
	g. *Roseburia*	Firmicutes	3.43	-0.26	0.02
	g. *Faecalibacterium*	Firmicutes	6.84	-0.31	0.006
	g. *Ruminococcus*	Firmicutes	7.48	0.26	0.02
Glucose	f. Peptostreptococcaceae	Firmicutes	0.66	-0.25	0.03
	g. *Dialister*	Firmicutes	1.06	-0.21	0.07
	c. Clostridia unclassified	Firmicutes	2.95	-0.21	0.06
Systolic BP	f. Catabacteriaceae	Firmicutes	0.43	0.36	0.001
	f. Peptostreptococcaceae	Firmicutes	0.66	-0.28	0.01
	g. *Bifidobacterium*	Actinobacteria	0.95	-0.24	0.04
	g. *Roseburia*	Firmicutes	3.43	-0.34	0.003
	g. *Bacteroides*	Bacteroidetes	8.59	-0.24	0.04
**Diastolic BP**	g. *Turicibacter*	Firmicutes	0.16	-0.34	0.003
	p. Firmicutes unclassified	Firmicutes	0.27	0.27	0.02
	g. [*Eubacterium*]	Firmicutes	0.27	0.29	0.01
	f. Catabacteriaceae	Firmicutes	0.43	0.32	0.007
	f. Lachnospiraceae	Firmicutes	0.59	-0.32	0.006
	g. *Dorea*	Firmicutes	0.90	0.28	0.01
	g. *Bacteroides*	Bacteroidetes	8.59	-0.28	0.01

*^1^Anthropometrics, biochemical markers and blood pressure collected at the end of the intervention: BMI, body mass index; LDL, low-density lipoprotein; HDL, high-density lipoprotein; BP, blood pressure.*

*^2^Some sequences were only affiliated to phylum (p.), class (c.), order (o.), family (f.), or genus (g.) levels.*

*^3^Abundance is shown for the composition of bacterial taxa in percentage (%).*

*^4^Non-parametric Spearman’s rank correlation coefficient.*

*^5^*P*-values are shown for the probability of the Spearman’s correlation.*

### Prediction of Functional and Metabolic Capacity of Gut Microbiota

**FIGURE 3:**
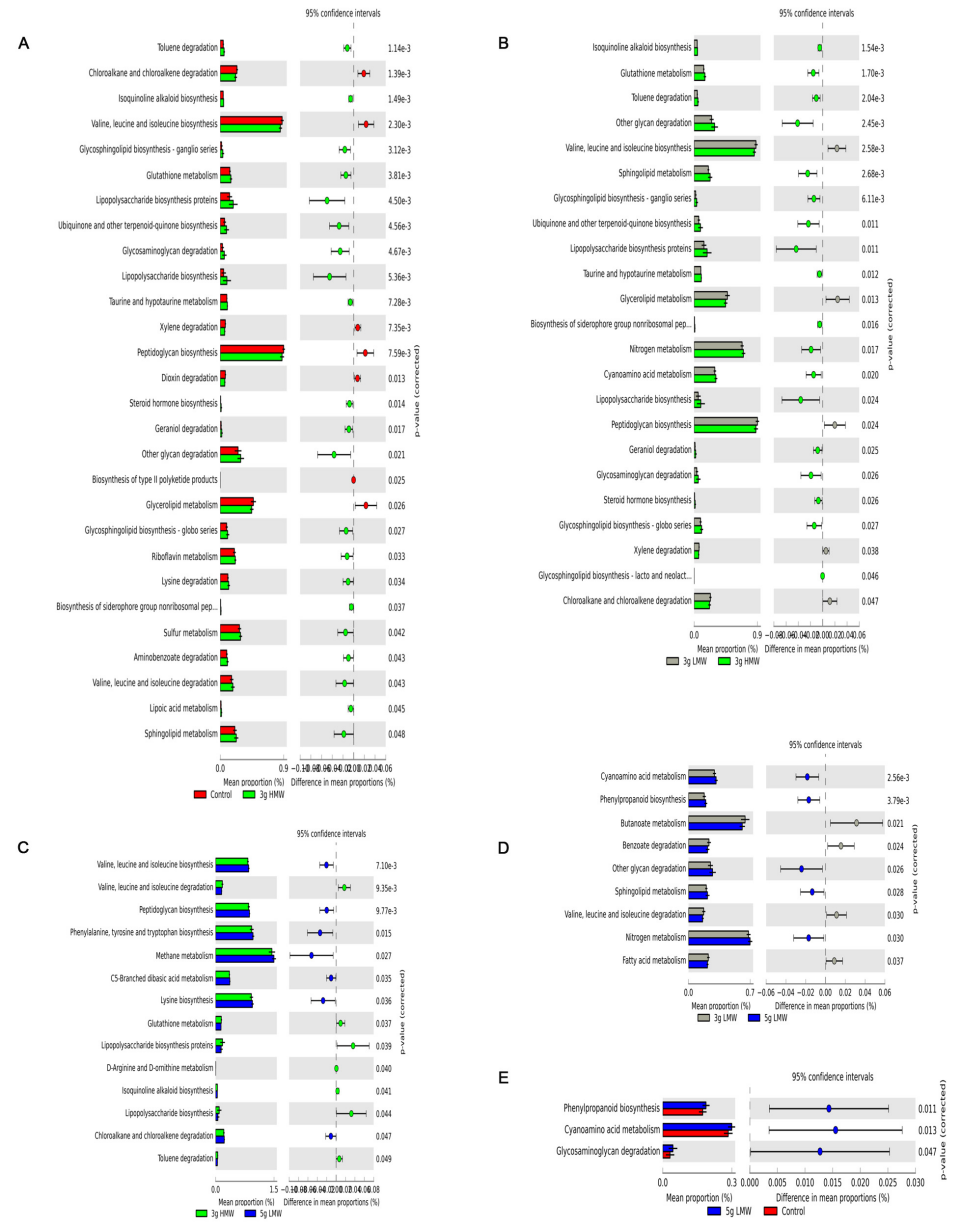
**Fold change in the number of 16S rRNA sequences annotated to KEGG level 3 orthologies representing the predicted metabolic capacity of the gut microbiome. (A)** 3 g HMW compared to control diet, **(B)** 3 g HMW compared to 3 g LMW diet, and **(C)** 3 g HMW compared to 5 g LMW diet, **(D)** 3 g LMW compared to 5 g LMW diet, and **(E)** 5 g LMW compared to control diet.

A total of 41 level 2 and 328 level 3 KOs were identified for the fecal microbiota (Supplementary Tables 2 and 3). Differences in metabolic potentials of gut microbiota among treatments were in line with OTU based differences observed with weighted UniFrac (**Figure 3**). Predicted shifts in glycerolipid metabolism and other glycan degradation were increased by 3 g HMW β-glucan compared with control (*P* = 0.026 and *P* = 0.021, respectively; **Figure 3A**). Same results for glycerolipid metabolism and other glycan degradation were observed when compare 3 g HMW vs. 3 g LMW β-glucan (*P* = 0.013 and *P* = 0.002, respectively; **Figure 3B**). However, although 5 g LMW β-glucan showed differences in weighted UniFrac compared with control, only three metabolisms (phenylpropanoid biosynthesis, cyanoamino acids metabolism, and glycosaminoglycan degradation) were predicted to be different using PICRUSt (**Figure 3E**), whereas no differences were observed between 3 g LMW β-glucan and control with both weighted UniFrac or PICRUSt (no difference to be graphed).

## Discussion

The main finding of this controlled feeding study was that gut microbiota were modulated in response to barley β-glucan consumption on a MW-dependent manner, and that the shifts in gut microbiota was correlated with reduced CVD risk factors. Specifically, consumption of 3 g/d HMW β-glucan resulted in increased abundance of p. Bacteroidetes and decreased abundance of p. Firmicutes, as did several of their respective taxa at the lower taxonomical levels (**Tables 2** and **3**). The shifts in g. *Bacteroides, Prevotella*, and *Dorea* were favorably correlated with an improved CVD risk factor profile, such as BMI, waist circumference, TG, blood pressure (**Table 4**). Consumption of 5 g/d LMW β-glucan only impacted the β-diversity but not the composition of gut microbiota compared to control (**Figure 1F**, **Tables 2** and **3**). Additionally, the consumption of 3 g/d LMW β-glucan failed to alter either the β-diversity or the composition of gut microbiota.

Previous *in vitro* fermentation and *in vivo* intervention studies have been conducted examining the ability of β-glucan to alter microbiota, however, conclusions were obscure leaving the breadth of the research inadequate (Crittenden et al., 2002; Dongowski et al., 2002; Hughes et al., 2008; Pieper et al., 2008; Mitsou et al., 2010). This was partially due to the inconsistencies in study design, experimental models and methodologies used for bacterial community analyses, e.g., quantitative PCR and fluorescence in site hybridization (FISH) that can only monitor selected members of the bacterial community. In the present research, we used a randomized controlled human trial that is considered gold standard for a clinical study. Furthermore, we employed 16S rRNA gene sequencing for bacterial community profiling, which is superior over previously used methods (AbuMweis et al., 2010b; Gilbert and Dupont, 2011).

Our results support that modulation in gut microbiota following barley β-glucan consumption is dependent on β-glucan MW as bacterial communities more profoundly shifted in response to diet containing 3 g HMW β-glucan. To date, there are few studies that have investigated the impact of physicochemical properties of β-glucan on microbiota composition. In an *in vitro* batch culture study, Hughes et al. (2008) assessed the impact of barley and oat derived β-glucan with different MW levels (average MW 130, 172, 243 kDa for barley β-glucan and 150, 230 kDa for oat β-glucan) on fecal microbiota using FISH and concluded that the *Bacteroides-Prevotella* group increased from baseline with all but the 243 kDa barley and 230 kDa oat substrates. Unfortunately, the β-glucan MW levels tested in the abovementioned study did not include ideal representatives of “high” MW β-glucan that could result in physiological benefits attributed to this compound (Tosh et al., 2008; Wolever et al., 2010). For instance, it has been shown that β-glucan with MW lower than 210 kDa fails to lower cholesterol levels (Wolever et al., 2010). Thus, the tested range of 150–243 kDa in the Hughes et al. (2008) study is considered very low MW and no true “high” MW level was examined. In our study, we selected the low and high MW β-glucan (288 and 1349 kDa, respectively) on the basis of cholesterol-lowering capability of this compound (Wolever et al., 2010).

The HMW β-glucan increases the viscosity of digesta (Tosh et al., 2010) in the GI tract, which is believed to slow down digesta transit time (Dikeman and Fahey, 2006; Lattimer and Haub, 2010). Consequently, the slower transit time may allow for prolonged exposure of fermentable compounds to microbial community (Koropatkin et al., 2012), possibly resulting in alteration of gut microbiota by HMW β-glucan observed in this study. Additionally, genes associated with glycosaminoglycan degradation and other glycan degradation were at higher abundance in the predicted metagenome with diet containing 3 g HMW β-glucan compared to the control (**Figure 3A**) indicating that certain members of the bacterial community, such as members of p. Bacteroidetes, had taken advantage of HMW β-glucan and perhaps other fermentable substrates in the digesta most likely due to increased exposure time for microbial fermentation. Lending support to this hypothesis, a recent study (El Kaoutari et al., 2013) examined the carbohydrate-digestive capacity of a simplified mini-microbiome, which was representative of human gut microbiome. Authors reported that members of p. Bacteroidetes had higher mean glycoside hydrolases (GHs) and polysaccharide lyases (PLs) genes per genome as well as signal peptide-containing GHs and PLs compared to the members of p. Firmicutes and any other bacterial phyla in the human gut. Authors further suggest that members of p. Bacteroidetes are likely the primary degraders of the many complex polysaccharides in the plant cell wall due to grater range of GHs and PLs that are not present or poorly represented in members of other gut bacterial phyla. This further supports our findings that members of p. Bacteroidetes have a competitive advantage in presence of HMW β-glucan that has higher glycosidic bonds compared to LMW β-glucan.

The observed changes in microbial community at the phylum level in response to 3 g/d HMW β-glucan were similar to the microbiota profile in “lean” individuals. Ley et al. (2006) described that the relative proportion of Bacteroidetes is decreased in obese people compared with lean people, and this proportion also increased with diet-induced weight loss. Similar to the microbiota profile of lean individuals, consumption of 3 g/d HMW β-glucan increased the Bacteroidetes and decreased the Firmicutes abundance, suggesting that consumption of HMW β-glucan may promote the healthy balance of microbiota in the gut (Ley et al., 2006). In the present study, weight loss was not observed in response to 3 g/d HMW β-glucan consumption most likely due to controlled study design, which intended to maintain body weight. To examine and interpret the potential weight loss effects of β-glucan consumption, further research with a specific study design aiming at the correlation between β-glucan consumption, microbiota shifts, and weight management is required.

The present intervention study showed that consuming 3 g/d of HMW β-glucan was able to significantly increase g. *Bacteroides* and moderately increase g. *Prevotella* in a short-term intervention of 35 days. The increased in g. *Bacteroides* were negatively correlated with BMI, waist circumferences and blood pressure (**Table 4**). Genus *Prevotella*, however, showed a negative correlation with TG. Additionally, g. *Dorea* that tended to be decreased by 3 g/d HMW β-glucan was positively associated with BMI, waist circumferences and diastolic blood pressure. Consumption of 3 g/d HMW β-glucan also decreased g. *Streptococcus* compared to the control. But this genus was not correlated with biochemical markers, anthropometric markers or blood pressure that were measured in the clinical trial. Altogether, these results suggested that consumption of 3 g/d HMW β-glucan for 35 days was able to alter the gut microbiota and the altered microbiota were correlated with a favorable shift in parameters associated with CVD. However, since these metabolic parameters were not changed significantly in the sampled subjects (*n* = 19), studies with greater sample size would be beneficial to further investigate the causality to see whether the altered gut microbiota improves the CVD risk factors.

In addition, predicted metabolic capacity of bacterial community (generated using PICRUSt) exhibited increased abundances of genes associated with glycerolipid metabolism with HMW β-glucan diet compared to control and LMW β-glucan diets, which has been associated with obesity and type 2 diabetes via energy homeostasis, appetite control, and insulin sensitivity (Prentki and Madiraju, 2008). These observations indicated that HMW β-glucan-induced gut microbial shifts may lead to reduced CVD risk and shifts the glycerolipid metabolism via an increased Bacteroidetes:Firmicutes ratio. That being said, we should take into account that PICRUSt predictions are based on known functions of the microbial communities present in the human GI tract obtained from the metagenomic studies. These predictions need to be confirmed using whole genome shotgun DNA (metagenomics) or RNA (metatranscriptomics) sequencing of analyzed samples.

Gut health in part is associated with proper richness, diversity and stability of gut microbiome. Recent research shows that individuals with low richness of microbiota are more vulnerable to obesity, insulin resistance and dyslipidemia compared to those with high richness of microbiota (Le Chatelier et al., 2013). In this study, the α-diversity of microbiota did not change by β-glucan consumption. β-diversity of microbiota, however, was impacted but the differences in β-diversity among treatments were significant when determined using weighted UniFrac distances rather than unweighted UniFrac distances, suggesting that β-glucan’s impact was through changing the abundance of certain members of the microbial community but not their presence and/or absence. As such, we believe that potential health benefit resulted from consumption of HMW β-glucan is via increase in the abundance of certain members of bacterial community but not community richness.

Overall, our results suggest that β-glucan with higher MW was able to alter the gut microbiota and the shifts observed were positively associated with an improved CVD risk factor profile. Although studies with large samples size are needed to confirm the relationship between the altered gut microbiota by β-glucan and the improved CVD health benefits, results from current study are reminiscent of the hypothesis that altering gut microbiota is responsible for the physiological benefits of HMW β-glucan consumption.

## Author Contributions

NA and PJ conceived and designed the experiment. NA, PJ, and YW conducted the clinical trial. YW performed lab analyses. YW, HT, and EK developed the bioinformatics and statistical models and analyzed the data. All authors drafted the manuscript. All authors carefully read and approved the final version of the manuscript.

## Conflict of Interest Statement

The authors declare that the research was conducted in the absence of any commercial or financial relationships that could be construed as a potential conflict of interest.

## Supplementary Material

FIGURE S1 **Principal coordinate analysis (PCoA) of weighted UniFrac distances among all treatments**.
