# Supplementary figures and images for: High Molecular Weight Barley β-Glucan Alters Gut Microbiota Toward Reduced Cardiovascular Disease Risk
Source: Front Microbiol. 2016 Feb 10;7:129. doi: 10.3389/fmicb.2016.00129 (PMC4748052; doi:10.3389/fmicb.2016.00129)

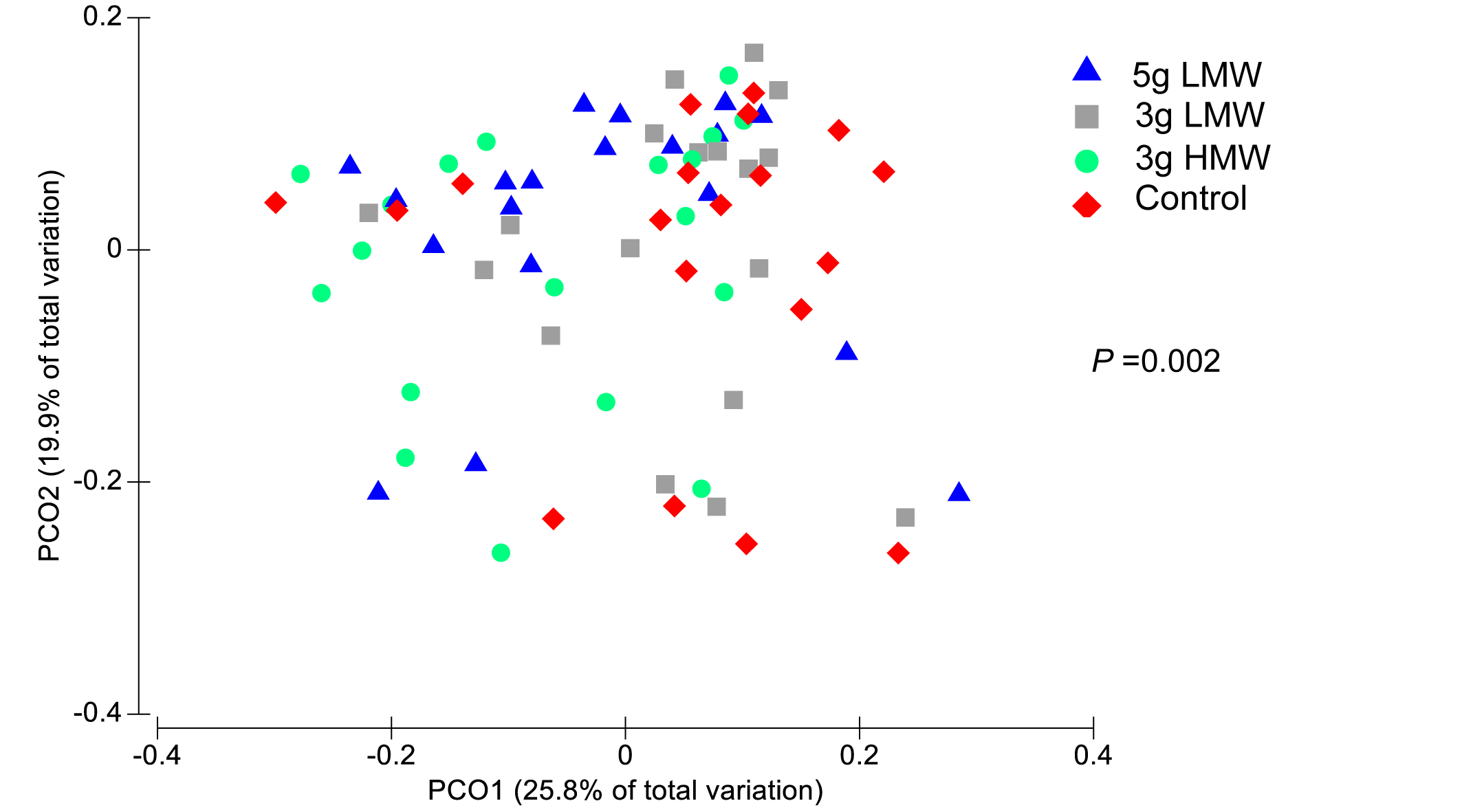

Supplement: FIGURE S1 — Principal coordinate analysis (PCoA) of weighted UniFrac distances among all treatments. [file Image_1.TIF]
